# Frequent Follow-Up of Delisted Liver Transplant Candidates Is Necessary: An Observational Study about Characteristics and Outcomes of Delisted Liver Transplant Candidates

**DOI:** 10.3390/jcm12185880

**Published:** 2023-09-10

**Authors:** Elnaz Payani, Dionysios Koliogiannis, Markus Bo Schoenberg, Dominik Koch, Daniela Eser-Valeri, Gerald Denk, Markus Rehm, Simon Schäfer, Ursula Ehmer, Andreas E. Kremer, Bruno Meiser, Jens Werner, Markus Guba, Nikolaus Börner

**Affiliations:** 1 Department of General-, Visceral- and Transplant Surgery, LMU University Hospital, 81377 Munich, Germany; elnaz.payani@med.uni-muenchen.de (E.P.); nikolaus.boerner@med.uni-muenchen.de (N.B.); 2 Transplant Center Munich, LMU University Hospital, 81377 Munich, Germanybruno.meiser@med.uni-muenchen.de (B.M.); 3 Faculty of Medicine, LMU University Hospital, 81377 Munich, Germany; 4 Department of Psychiatry, LMU University Hospital, 81377 Munich, Germany; 5 Department of Medicine II, LMU University Hospital, 81377 Munich, Germany; 6 Department of Anesthesiology, LMU University Hospital, 81377 Munich, Germany; 7 Department of Anesthesiology, Carl von Ossietzky University Oldenburg, 26121 Oldenburg, Germany; 8 Department of Medicine II, Technical University (TU) Munich Klinikum Rechts der Isar, 81675 Munich, Germany; 9 Department of Medicine I, Friedrich-Alexander University Erlangen-Nürnberg, 91054 Erlangen, Germany; 10 Department of Gastroenterology and Hepatology, University Hospital Zurich, Rämistrasse 100, 8091 Zürich, Switzerland

**Keywords:** liver transplantation, liver transplant waiting list, delisting, transplant conference

## Abstract

This observational study focuses on the characteristics and survival of patients taken off of the liver transplant waiting list. Assessment of post-delisting survival and a frequent follow-up of patients after delisting are important keys to improve the survival rate of patients with liver failure after being delisted. Within this study, delisted liver transplant candidates were divided into the following groups: (1) “too good” (54%) or (2) “too sick” (22%) for transplantation, (3) adherence issues (12%) or (4) therapy goal changed (11%). The 5-year survival after delisting within these groups was 84%, 9%, 50%, and 68%, respectively. Less than 3% of the delisted patients had to be relisted again. The clinical expert decision of the multidisciplinary transplant team was sufficiently accurate to differentiate between patients requiring liver transplantation and those who were delisted after a stable recovery of liver function. The assessment of post-delisting survival may serve as a complementary metric to assess differences in center practices and to estimate cumulative post-delisting mortality risk.

## 1. Introduction

Liver transplantation (LT) is the only definitive therapy for end-stage liver disease and is considered as the standard of care for suitable candidates. Management of patients on the LT waiting list is an important key to improve the result of transplantation and its survival rate. After any changes in the patient’s status or in the course of diseases, it is important to decide which patients should remain on the LT waiting list and which should drop out, in order to improve the short- and long-term outcomes of LT, as well as the interests of patients [1].

Although the criteria for listing of liver transplant candidates is well-established, the criteria for delisting are poorly defined. The decision to delist a patient relies on the clinical consensus of all members of a transplant team and carries profound clinical, social, and ethical implications. In the Eurotransplant region, approximately 15% of patients who are listed for liver transplantation are subsequently delisted without being transplanted [2]. The reasons for delisting are often multifaceted, but typical clinical trajectories can be identified. The main reasons for delisting include liver function recompensation; becoming unfit for liver transplantation; tumor progression, such as hepatocellular carcinoma (HCC); adherence issues often related to failure to abstain from alcohol; and changes in therapy goals. Especially following the recompensation of liver function to a level where transplantation is no longer necessary, questions arise regarding the stability of the condition. When delisting occurs due to unfitness, it is crucial to monitor the delisting criteria to avoid unjustly denying transplantation access to a patient who, despite his/her unfitness, could still benefit from transplantation. The criteria for a final delisting are unclear and are significantly influenced by the center’s policy [3]. Moreover, prior studies have highlighted the impact of socioeconomic disparities on delisting decisions and have focused on the question of which predictors can be used to determine whether patients can recover from liver decompensation [3,4,5,6]. This latter aspect is gaining prominence, particularly with effective therapies, such as those for chronic hepatitis C [6].

Herein, we present a study focused mainly on the outcomes of patients who have been delisted for liver transplantation. Understanding the outcomes of these patients may help improve survival on the waiting list and after delisting. Our study aims to further characterize those patients who have been delisted and to provide a stronger basis to estimate the cumulative transplant benefit for individual patients.

## 2. Materials and Methods

### 2.1. Study Population

In this current retrospective multicenter observational study, data from all patients listed for liver transplantation who were subsequently delisted between 2009 and 2019 were collected from four transplant centers (LMU Klinikum, Munich, Germany and collaborating centers, Klinikum Rechts der Isar, TU Munich, University Clinic, Erlangen, Germany). Demographic and clinical data of the patients at the time of listing and delisting, as well as the survival rate after delisting, were collected from a prospectively maintained database and the Eurotransplant database. Data were collected from the time of listing until the last follow-up in 2020 or until the patient died. The study protocol has been approved by the local ethics committee (number 19-395).

### 2.2. Listing and Delisting Criteria/Policy

The decision to list or delist a patient was made by the interdisciplinary transplant conference, which comprised a transplant surgeon, hepatologist, anesthesiologist/intensivist, and psychiatrist. Patient inclusion on the waiting list followed the guidelines of the German Medical Association (“Bundesaerztekammer”). The decision to actively list a patient was assisted by criteria proposed by Luo et al. for determining transplant benefits [7]. Simplified, these were end-stage liver disease patients with a labMELD score of ≥15, as well as those with severe complications of liver cirrhosis, such as esophageal variceal hemorrhage, ascites, or HCC within accepted transplant criteria. In addition, patients with other life-limiting liver diseases that can appropriately be treated with liver transplantation were also included. An estimated probability of success of at least 50% 5-year survival was applied as a benchmark for listing.

Patients were delisted if (1) there was no longer an indication for transplantation due to stable improvement in liver function; (2) the patient’s condition was too poor for transplantation (sepsis, infection, or the need for ICU admission), or tumor progression precluded transplantation; (3) insurmountable adherence problems were present; or (4) there was a change in therapy goals, and liver transplantation was no longer pursued for various reasons. In each scenario, the delisting of a patient was confirmed by the interdisciplinary transplantation conference following an observation period in “not transplantable (NT)” status, which designates patients as temporarily not transplantable.

### 2.3. Variables and Statistical Analysis

Data included gender, age at listing and delisting, height, weight, ABO blood group, date of listing and delisting, listing indications, labMELD score at listing and delisting, date of death, duration on waiting list, reasons for removal, relisting date, and insurance status. Categorical variables were described with frequency of occurrence and continuous variables with medians and interquartile ranges (IQRs). To compare the characteristics in different delisted categories, we used the Kolmogorov–Smirnov test, ANOVA, and the Kruskal–Wallis test. Overall survival was defined as the primary outcome parameter. To estimate the 5-year survival in different groups of delisted candidates, we used the Kaplan–Meier method. Delta-MELD was analyzed for association with the outcome using C-Statistic Analysis. A *p*-value less than 0.05 was considered as statistically significant. Statistical analysis and diagrams were performed with Microsoft Excel v2010 and SPSS for Windows v24 (IBM Corp, Armonk, NY, USA). The graphic abstract was provided using www.canva.com (accessed on 23 July 2023).

## 3. Results

### 3.1. Study Population and Patients Characteristics

A total of 910 patients were listed between 2009 and 2019: 592 were transplanted, 121 died on the waiting list, 1 patient was lost to follow-up, and 196 patients were delisted and included in the study. The majority were male (62.8% vs. 37.2% female). The median age at the time of listing was 52 (IQR: 14.25), and at the time of delisting, it was 55 (IQR: 16). The majority had public health insurance (84.7% vs. 15.3% private insurance). The median BMI in the entire population was 24 (range 14–43). The duration of waiting time on the transplantation waiting list ranged from 0 to 184 months, with a median of 21 months (IQR: 41). The most common blood group among the patients was A (50%), followed by O (33.7%), B (10.7%), and AB (5.6%). The median labMELD score at listing and delisting were 13 (IQR: 7) and 11 (IQR: 7), respectively. Patients were further categorized, based on the leading reason for delisting, into the following study groups: “too good”, *n* = 106; “too sick”, *n* = 44; adherence problems, *n* = 24; and therapy goal change, *n* = 22. Patients’ characteristics are detailed in Table 1. There was no significant statistical difference in patients’ characteristics between the compared groups.

### 3.2. Primary Indications and Reasons for Delisting

In the entire study population, the most common primary indications for liver transplantation were alcoholic cirrhosis without HCC (*n* = 58, 29.6%), followed by HCC (*n* = 45, 22.9%), chronic viral hepatitis without malignancy (*n* = 24, 12.2%), metabolic and genetic liver diseases (*n* = 18, 9.1%), and cholestatic liver diseases (*n* = 17, 8.6%). “Too good” for liver transplantation was the most common reason for delisting (54%). The most prominent subgroup within this category is patients with alcoholic liver disease (ALD) without HCC whose liver function recovered under alcohol abstinence (see Table 1). Recompensation in this group of patients was accompanied by a significant improvement in the median labMELD score from 15 to 11. The second largest group was patients who were “too sick” for transplantation (22%). This group includes HCC patients with tumor progression beyond the accepted transplant indication and patients who became unfit for transplantation because of comorbidities or sepsis or multiple organ failure. Overall, 12% of the patients were delisted due to adherence problems, most of them due to relapse into abusive drinking behavior. In this group, there was no improvement in the labMELD score from the time of listing to delisting. Except for the waiting time, no significant differences were observed among demographic data and characteristics in these four groups. The median waiting time until delisting was 21 months. LT candidates who were removed from the list after clinical recovery (“too good” group) had been on the waiting list for 0–184 months (median waiting time = 28 months), patients in the “too sick” group for 0–78 months (median waiting time = 7 months), candidates with adherence problems for 0–91 months (median waiting time = 18 months), and candidates removed due to a change in therapeutic goal for 1–118 months (median waiting time = 24 months). A significant difference in the median waiting times of delisted candidates was observed among the four groups (*p* = 0.006).

### 3.3. Outcome after Delisting

**Figure 1:**
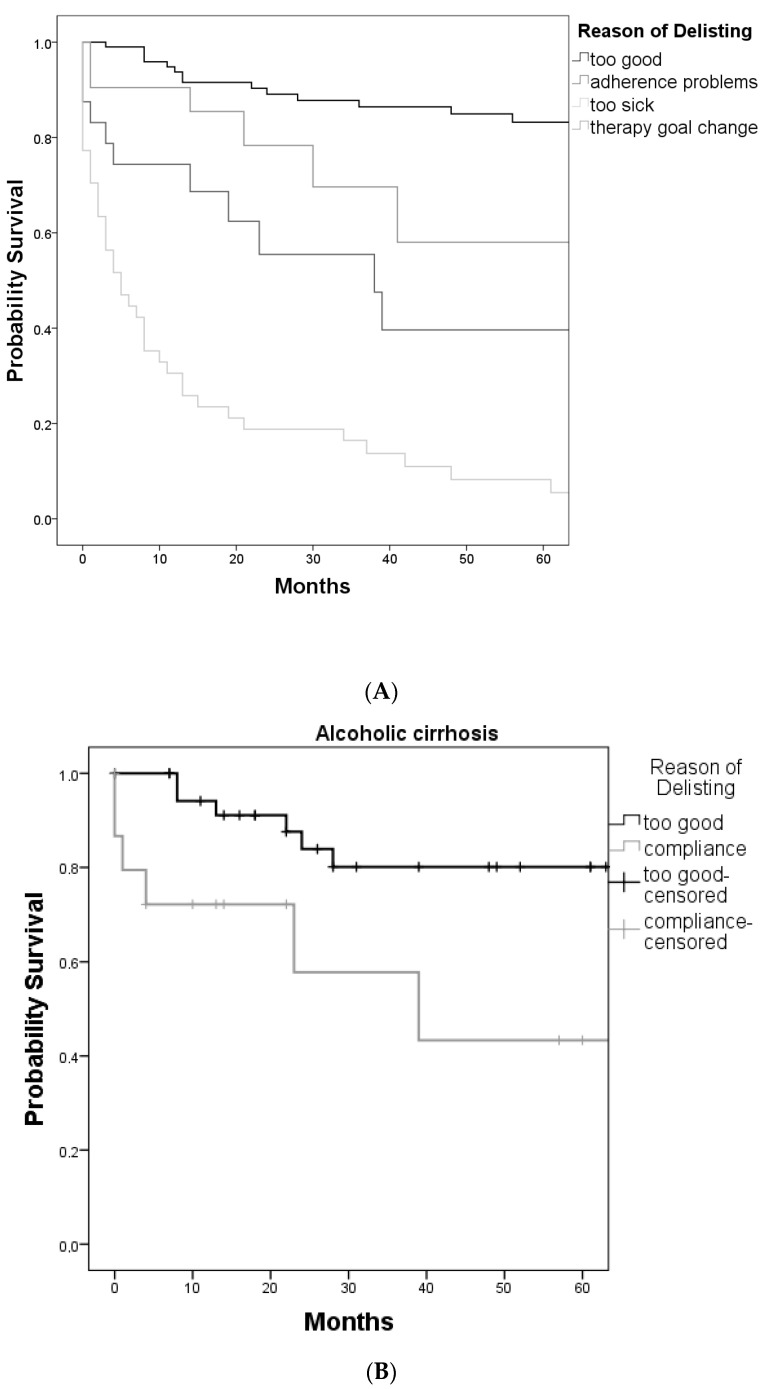
(**A**) Kaplan–Meier estimate of patient survival after delisting according to delisting categories. (**B**) Kaplan–Meier estimate of post-delisting survival in ALD patients. Survival of patients delisted as “too good” is compared to patients delisted for adherence problems.

The post-delisting survival of the four study groups is shown in Figure 1A. The 5-year overall survival of all patients, regardless of the delisting reason, was 61.2%. The 1-year, 2-year, 3-year, and 5-year overall survival rates for the patients in the delisting category “too good” were 97%, 92%, 89%, and 84%, respectively. Within this group, the 5-year survival was 100% for patients recovered from HCV, patients with autoimmune hepatitis, and patients with acute liver failure; 91% for patients with cholestatic liver disease; 80% after recovering from HBV; 76% after recovering from decompensated alcoholic liver disease; and 60% for HCC patients. The 1-year, 2-year, 3-year, and 5-year survival rates of patients categorized as “too sick” for transplantation were 33%, 19%, 17%, and 9.%, respectively. The median survival after delisting in this group was 4 months. Patients who had been listed because of acute liver failure, HCV, or cholestatic liver disease and later were delisted as “too sick” had a 5-year survival of 0%; patients with alcoholic liver disease or metabolic/genetic liver disease 25%; and HCC 8%. In this group, we observed a 0% 5-year survival in patients with tumor progression and a 16% 5-year survival in patients with other causes of decompensation. There was no significant difference observed here (*p* = 0.18). The 1-year, 2-year, 3-year, and 5-year survival rates in patients with adherence problems were 75%, 62%, 57%, and 40%, respectively. The median survival after delisting was 9 months (see Figure 1B).

In patients who were delisted due to a change in therapeutic goal, the 1-year, 2-year, 3-year, and 5-year survival rates were 92%, 79%, 70%, and 68%, respectively. The median interval from delisting to death was 21 months (range 1–71 months).

A comparison between the survival rates of patients who were initially listed due to alcoholic liver cirrhosis and were delisted either after liver function recovery or due to non-compliance reveals a significant difference between the survival rates in these two groups. The 1-year, 2-year, 3-year, and 5-year survival rates for patients with alcoholic liver cirrhosis who were delisted after liver function recovery as “too well for LT” were 94%, 84%, 80%, and 80%, respectively. These rates were 72%, 54%, 54%, and 43% for patients with alcoholic liver cirrhosis who were delisted due to non-compliance (*p* = 0.003) (Figure 1B).

### 3.4. Relisting after Delisting

In total, 5 of 196 patients (2.6%) were relisted after delisting. Among them, three patients were relisted after being delisted for recompensation due to renewed deterioration. One patient was relisted 15 days after the initial delisting due to tumor progression, while another patient was relisted once adherence problems were resolved.

## 4. Discussion

This paper focuses on the outcomes of patients who have been removed from the liver transplant waiting list. To our knowledge, this is the first study that has systematically tracked patient survival following delisting [8].

The results of our study show that the survival of patients classified as “too good” during the waiting time was excellent after delisting, with 60–100% 5-year survival, depending on the underlying disease. Most of these patients showed delisting survival rates far above what could have been expected after liver transplantation. This was particularly notable among HCV patients who had undergone successful antiviral treatment. The potential of novel antiviral therapies to avoid liver transplantation in wait-listed patients has been noted in several recent studies [9]. Based on our findings, the majority of delisted patients in the “too good” category might not have gained significant benefits from liver transplantation. As a result, the imperative to identify individuals on the waiting list who are likely to recover to a state where transplantation becomes unnecessary becomes even more pronounced. The Toronto group has identified MELD <20 and a serum albumin at the time of listing as the only independent predictors for successful delisting for patients with alcoholic cirrhosis [3]. The probability of recompensation was 70% when both factors were present at listing. However, they also note that a quarter of patients who received a living-donor liver transplant with a median waiting time of 2 months met these criteria. It is, therefore, very likely that some of the transplanted patients would have recovered spontaneously without transplantation with sufficient waiting time. With this consideration in mind, it is our policy to monitor “borderline” patients over two years in status “NT” to evaluate their potential to recover. We have applied this practice not only to patients with decompensated liver function as the primary indication for liver transplantation, but also to HCC patients with a complete response to locoregional bridging therapy [10]. For this group, we have found almost similar survival as compared to patients who were transplanted, which emphasizes the point to observe patients to follow their tumor biology or self-healing capacity. A relisting rate of less than 3% in the patients delisted for “too good” suggests that expert decision making by a multidisciplinary transplant team with an adequately long observation time can differentiate sufficiently precisely between patients requiring liver transplantation and those who are better to be delisted.

Not surprisingly, the survival of patients delisted for being “too sick” was dismal. This group includes patients who either experienced tumor progression or were unfit because of their clinical condition or comorbidities. Both subgroups justify separate consideration. In patients with progressive HCC, the decision to delist is warranted for oncological reasons [11] Accordingly, patients with HCC progression died within a very short time after delisting under palliative therapy. In the group of patients considered to be unfit for transplantation, however, the unpleasant question arises whether some of these patients were unjustifiably denied a transplant and, thus, a chance of survival. Due to the prevailing shortage of organs, decisions must be made by the transplantation team to ensure that the available donor organs are used efficiently [12,13]. Because of this inevitability, patients with a very low chance of success with transplantation are being directed to alternative therapeutic concepts. It is particularly important for the transplant team’s decision-making process to avoid the influence of external metrics on center performance. In this context, Kwong et al. have shown significant variations of US centers’ risk aversion potentially caused by quality metrics and unintended network effects [14]. In addition, there may be a systematic bias toward certain patient groups. For example, Cullaro et al. showed a significant sex-based disparity in the delisting of patients who were “too sick” for liver transplantation [5]. In the same context, Krystal et al. showed an influence of payer status on the delisting of patients on the liver transplant list [6]. Although we cannot completely rule out the possibility of a systematic bias, we could not find any such bias in the factors we analyzed.

Withholding a life-sustaining liver transplantation because of non-adherence has always been the subject of extensive ethical debates. Also, the ability to adhere depends significantly on socioeconomic status and the assistance provided. Furthermore, adherence is not a stable characteristic and can fluctuate significantly over time. The vast majority of adherence problems in patients on the liver transplant waiting list occur in the context of expected alcohol abstinence. Since in Germany, as in most other countries, a minimum of 6 months of ETG-controlled alcohol abstinence is required, the margin of decision making for the transplant team is limited here. For insurmountable alcohol adherence problems, patients are delisted regardless of urgency. Accordingly, our data show that in patients who adhere to the abstinence rule, liver function can recompensate to a point where liver transplantation is no longer needed. Patients delisted after recovery had an excellent post-delisting 5-year survival of more than 80%. On the contrary, the post-delisting 5-year survival of patients with ALD who were delisted for non-adherence was a little below 50%. Nearly half of ALD patients, however, survive longer term, even after delisting, despite continued alcohol abuse.

Finally, we have followed a not well-defined group of patients, where it was decided during the waiting period to change the therapeutic goal. Formally, these patients continued to have a transplant indication, but, besides that, had other very understandable reasons to no longer pursue liver transplantation. Among those were older age and a low likelihood of a timely organ allocation. Surprisingly, these patients had an acceptable survival after delisting, so the decision not to have transplantation was probably the right one for the majority of these patients.

The results of this observational study have to be interpreted with caution. As noted above, delisting decisions are not standardized and can vary substantially from center to center, based on different clinical assessments by the transplant team, external quality benchmarks, and other confounding factors. A comparison between the survival rates of delisted patients and those patients who died on the waiting list can be useful to assess the post-delisting survival. It is a limitation of our study that these data were not available.

In summary, we showed that the clinical decision of the multidisciplinary transplant team was sufficiently accurate to differentiate between patients requiring liver transplantation and those who were delisted after a stable recovery of liver function. Understanding the post-delisting survival of specific patient groups can help the interdisciplinary transplant team to estimate the individual transplant benefit throughout various phases of transplant-related care. To better understand the outcomes after delisting on a larger scale and to identify factors associated with mortality, prospective studies with long-term follow-up are necessary.

## Figures and Tables

**Table 1:** Patients’ characteristics.

	Overall (*n* = 196)	“Too Good” (*n* = 106)	“Too Sick” (*n* = 44)	Adherence Problems (*n* = 24)	Therapy Goal Change (*n* = 22)	*p*-Value
Age at listing (years) *	52 (14.25)	52 (16.5)	54 (13.25)	48 (13)	54 (13.5)	0.32
Age at delisting (years) *	55 (16)	55 (17)	55 (13.25)	50.5 (14)	57 (14)	0.62
Gender (m/f)	123/73	63/43	35/9	12/12	13/9	0.055
BMI at listing (range)	24 (14–43)	24 (14–39)	24 (19–43)	24 (15–37)	24 (18–35)	0.74
Blood group (%)						0.49
O	66 (33.7)	35 (33)	16 (36.4)	7 (29.2)	8 (36.4)	
A	98 (50)	52 (49.1)	20 (45.5)	17 (70.8)	9 (40.9)	
B	21 (10.7)	12 (11.3)	5 (11.4)	0 (0)	4 (18.2)	
AB	11 (5.6)	7 (6.6)	3 (6.8)	0 (0)	1 (4.5)	
Insurance status (*n*/%)						0.84
private	30 (15.3)	18 (16.9)	5 (11.4)	4 (16.7)	3 (13.6)	
public	166 (84.7)	88 (83.1)	39 (88.6)	20 (83.3)	19 (86.4)	
Waiting time (months) *	21 (41)	28 (46)	7 (18)	18 (25)	24 (38)	
_lab_MELD *						
at listing	13 (7)	14 (9)	12 (6)	16 (3)	12 (8)	0.26
at delisting	11 (7)	10 (5)	14 (7)	17 (9)	12 (7)	0.80
Etiology of LD (%)						
ALF	14 (7.1)	11 (10.4)	2 (4.5)	0	1 (4.5)	
ALD *w*/*o* HCC	58 (29.6)	37 (34.9)	4 (9.1)	15 (62.5)	2 (9.1)	
HBV *w*/*o* HCC	6 (3.1)	4 (3.8)	0	0	2 (9.1)	
HBV/HDV *w*/*o* HCC	2 (1)	1 (0.9)	1 (2.3)	0	0	
HCV w/o HCC	16 (8.2)	7 (6.6)	6 (13.6)	2 (8.3)	1 (4.5)	
HCC in ALD	18 (9.2)	5 (4.7)	10 (22.7)	1 (4.2)	2 (9.1)	
HCC in HBV	6 (3.1)	1 (0.9)	5 (11.4)	0	0	
HCC in HCV	11 (5.6)	2 (1.9)	6 (13.6)	2 (8.3)	1 (4.5)	
HCC unknown	10 (5.1)	4 (3.8)	2 (4.5)	0	4 (18.2)	
AIH *w*/*o* HCC	5 (2.6)	4 (3.8)	0	0	1 (4.5)	
Cholestatic LD	17 (8.7)	11 (10.4)	2 (4.5)	2 (8.3)	2 (9.1)	
Cryptogenic LC	8 (4.1)	3 (2.8)	2 (4.5)	1 (4.2)	2 (9.1)	
Metabolic/genetic	18 (9.2)	11 (10.4)	4 (9.1)	1 (4.2)	2 (9.1)	
Other	7(3.5)	5 (4.7)	0	0	2 (9)	

* median/IQR. ALF: acute liver failure, ALD: alcoholic liver disease, HBV: hepatitis B virus, HCV: hepatitis C virus, HCC: hepatocellular carcinoma, AIH: autoimmune hepatitis, w/o: without.

## Data Availability

The data that support the findings of this study are available on request from the corresponding author, Markus Guba. The data are not publicly available due to the privacy of the research participants and ethical restrictions.
